# A new era for *Animal Welfare*

**DOI:** 10.1017/awf.2023.3

**Published:** 2023-01-24

**Authors:** Huw Golledge, Birte Nielsen

**Affiliations:** Editors-in-Chief, Animal Welfare, Universities Federation for Animal Welfare

**Keywords:** animal welfare, publishing, open access, journal, academic publishing, open science

With the publication of this editorial *Animal Welfare* enters a new era in a new format: the Journal is now Gold Open Access with continuous online-only publication of high-quality animal welfare research.

*Animal Welfare*, the longest continuously published animal welfare journal is the scientific journal of the Universities Federation for Animal Welfare (www.ufaw.org.uk)(UFAW), a non-profit society dedicated to the promotion of animal welfare science. As a society journal, *Animal Welfare* is dedicated to the dissemination of evidence-based animal welfare information, which is central to UFAW’s mission. The transition to Gold Open Access means the Journal will now do an even better job of fulfilling that mission. Like UFAW, our new publishing partner Cambridge University Press https://www.cambridge.org/ (CUP) is a non-profit organisation, and together we have transitioned the Journal to a sustainable, fully open access model that serves the unique needs of the animal welfare science community. This means we can continue to publish high quality animal welfare research whilst ensuring that it is accessible to everyone as quickly and widely as possible. Since the mission of UFAW and the Journal is to bring about improvements in animal welfare via a better understanding of animal welfare problems and their solutions, it is crucial that anyone who can make use of the research we publish can access it freely. We are delighted that this is now the case. The transition from a printed journal to online-only publication will also reduce our environmental impact, whilst reducing the delay between acceptance of papers and publication.

The editorial team https://www.cambridge.org/core/journals/animal-welfare/information/about-this-journal/editorial-board are very excited about the move to open access, online publishing. Following the transition, we will continue to work on improving the submission and publishing process. As well as a brand-new cover design, one recent development is a change to format-neutral initial submissions of manuscripts. This means that authors do not need to format their article to the journal style at this initial stage, and figures and tables can be kept in their original locations in the text, which also makes reviewing easier. This is just one example of how we are working to make the Journal user-friendly and accessible to improve the experience for authors and reviewers.

Another change is an expansion of the section on ethical considerations, included in all experimental papers, where we encourage authors to describe in detail the ethical considerations concerning of their use of animals (or humans) in research. Whether or not an ethics committee has approved the study, we would like to hear more about the harm-benefit assessments made by the researchers, and a description of how the 3Rs and other ethical principles were implemented. For more details on this, see the section on Ethical Considerations in the author instructions https://www.cambridge.org/core/journals/animal-welfare/information/author-instructions.

At UFAW, as well as ensuring that anyone who needs to can access the latest animal welfare research we also want to make sure that everyone has the chance to publish their research in *Animal Welfare*, regardless of their ability to pay Article Processing Charges https://www.cambridge.org/core/journals/animal-welfare/information/author-instructions/fees-and-pricing (APCs). Some authors will be covered by one of CUP’s Transformative Agreements https://www.cambridge.org/core/services/open-access-policies/read-and-publish-agreements (sometimes known as Read-and-Publish deals) allowing academics at many institutions to publish without directly paying an APC. For authors who are not covered by a transformative agreement, and do not have funding for APCs, the Journal can offer discretionary waivers of the APC as well as automatic full or partial geographic waivers to authors based in a lower income country which is on the Research4Life https://www.research4life.org/access/eligibility/ list.

As before, any profits made by the Journal will continue to be ploughed back into the animal welfare work carried out by UFAW, in the form of support for research and dissemination of new knowledge.

Alongside this editorial we are publishing the first papers under our new publishing model. These include studies on highly topical areas, that have been the subject of debate for decades such as the control of rats and mice when they come into conflict with humans and emerging areas such as the animal welfare challenges of farming insects for food (Barrett & Fischer 2023; De Ruyver *et al.* 2023.

As a society journal, we hope that you will continue to support *Animal Welfare* in its new format, as authors, reviewers, members of the editorial board – and as readers.
